# Quantitative Source Apportionment of Potentially Toxic Elements in Baoshan Soils Employing Combined Receptor Models

**DOI:** 10.3390/toxics11030268

**Published:** 2023-03-14

**Authors:** Chunyu Dong, Hao Zhang, Haichan Yang, Zhaoxia Wei, Naiming Zhang, Li Bao

**Affiliations:** 1 Yunnan Agricultural University, Kunming 650201, China; 2 Yunnan Laboratory of Improvement of Soil Fertility and Pollution Remediation, Kunming 650201, China

**Keywords:** cultivated soils, potentially toxic elements, receptor model, source apportionment, soil pollution

## Abstract

Arable soils are crucial for national development and food security; therefore, contamination of agricultural soils from potentially toxic elements (PTEs) is a global concern. In this study, we collected 152 soil samples for evaluation. Considering the contamination factors and using the cumulative index and geostatistical methods, we investigated the contamination levels of PTEs in Baoshan City, China. Using principal component analysis, absolute principal component score-multivariate linear regression, positive matrix factorization, and UNMIX, we analyzed the sources and quantitatively estimated their contributions. The average Cd, As, Pb, Cu, and Zn concentrations were 0.28, 31.42, 47.59, 100.46, and 12.36 mg/kg, respectively. The Cd, Cu, and Zn concentrations exceeded the corresponding background values for Yunnan Province. The combined receptor models showed that natural and agricultural sources contributed primarily to Cd and Cu and As and Pb inputs, accounting for 35.23 and 7.67% pollution, respectively. Industrial and traffic sources contributed primarily to Pb and Zn inputs (47.12%). Anthropogenic activities and natural causes accounted for 64.76 and 35.23% of soil pollution, respectively. Industrial and traffic sources contributed 47.12% to pollution from anthropogenic activities. Accordingly, the control of industrial PTE pollution emissions should be strengthened, and awareness should be raised to protect arable land around roads.

## 1. Introduction

The expansion of cities and rapid development of modern agriculture have led to increased environmental pollution in cultivated soils. Some of the contaminants of greatest concern are potentially toxic elements (PTEs) [1,2], which not only destroy the quality of cultivated land, but also they indirectly or directly cause damage to human health [3,4]. Therefore, remediation of arable soil PTE contamination has become one of the most urgent problems in environmental science. However, PTEs in soils do not originate from human activities such as mining, metal smelting, pesticide/fertilizer usage, or automobilism [5,6,7]. Furthermore, several researchers have shown that the mineralization of soil parent material is the main cause of PTE enrichment [8,9].

Yunnan province has a complex geological structure and rich mineral resources. According to a 2020 report on the quality of arable land in the Baoshan region, medium-grade cultivation was performed on 263,500 ha of low-grade cultivated land, which accounted for 79.31% of the cultivated land area [10]. Currently, soil conditions are poor and heavy metal pollution is becoming an increasingly significant problem in the area [11,12,13,14,15]. Moreover, the PTE background value in Baoshan is significantly higher than the national average, making PTE pollution a serious hazard in the region [16,17]. 

Zhang et al. have found severe PTE enrichment in soils around the Baoshan mining area and have reported the soil to be at risk [18]. Other factors affecting Baoshan soil PTE enrichment include geogenesis and carbonate and basalt parent material differentiation. The Baoshan agricultural environmental protection monitoring station that evaluates the quality of farmland soil indicated that the arable soil in the study area is contaminated to varying degrees by different sources, the most prominent ones being Cd, Pb, As, Cu, and Zn PTEs [19,20,21]. Importantly, the quality and safety of arable soil determines the quality and safety of agricultural products. Cicchella et al. has recommended paying attention to the nature and sources of contaminants [22]. Still, previous studies have only evaluated the risk of soil contamination in Baoshan and have not distinguished the sources of contamination [23]. Thus, we must understand the sources of PTE pollution in arable land to protect it [24,25].

The methods of pollution source analysis are divided mainly into source identification and source apportionment. Scholars are increasingly using receptor models for source analyses, with the most widely used ones being principal component analysis (PCA), absolute principal component score–multivariate linear regression (APCS-MLR), UNMIX, and positive matrix factorization (PMF). Li et al. used PCA to determine potentially toxic elements in Gansu cropland soils; they found three sources of contamination and evaluated soil data [26]. The same group also used APCS-MLR to analyze the PTE sources in urban farmland. They combined the total hazard index (THI) and total carcinogenic risk (TCR) and found that APCS-MLR analyzed both the pollution sources and pollution status [27]. This model only required the composition of the receptor emission source and did not require the accurate source component spectrum data. Still, the estimated source component spectrum and contribution values were often observed. Chen et al. combined UNMIX with PMF for source analysis of heavy metal data in a suburban area of Kaifeng and found good agreement between the two models for source assignment [28]. The UNMIX model overcomes the drawback of negative source contribution, which does not require prior knowledge of the number of sources and identifies these sources via different source identifiers to infer them. However, it cannot determine the source components, contribution rates, or contribution values. Liu et al. used PMF to determine the pollution sources in a farmland in Wenzhou, Zhejiang Province, and combined it with multivariate statistical methods, which demonstrated the feasibility of PMF models for source analysis [29]. Lv used APCS/MLR and PMF, and both provided three identical sources and were consistent with the mapping of parent material distribution [30]. PMF avoids negative values in the results, thus providing interpretability and clear physical meaning to the obtained source component spectra and source contributions. In addition, PMF does not require the measurement of source component spectra and uses error estimates for each individual data point to deal with missing and imprecise data [31].

Therefore, the main objectives of this study were to determine the concentrations of five soil PTE elements (Cd, Pb, As, Cu, and Zn) and to investigate the sources of PTEs in soil samples from Baoshan cropland. This would allow us to better understand the specific sources of PTEs contamination in Baoshan cropland and to provide a reliable basis for improving pollution in the area. Specifically, our aims were to: (1) investigate the pollution status of five soil PTEs in Baoshan soil; (2) map the distribution of these soil PTEs using GIS; (3) identify the sources of contamination using PCA, APCS-MLR, PMF, and UNMIX models; and (4) compare the contributions of the four receptor models.

## 2. Materials and Methods

### 2.1. Study Area

**Figure 1:**
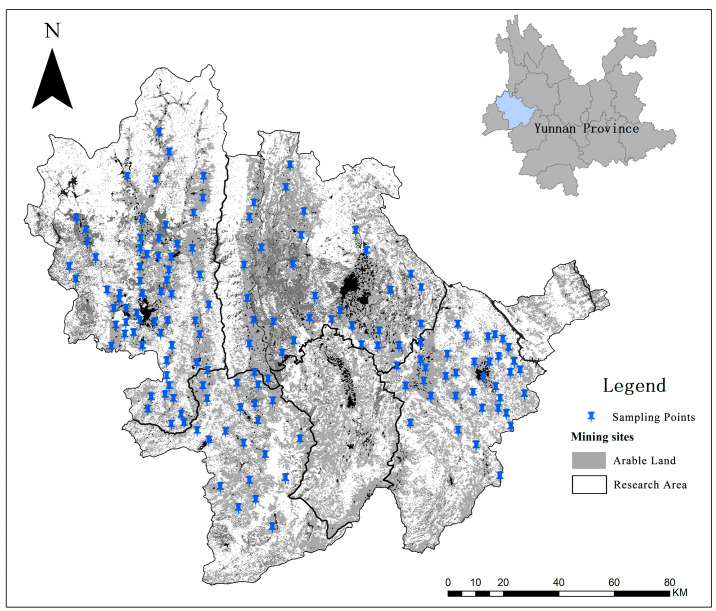
Sampling locations of the study area.

Baoshan is located in the western part of the Yunnan Province of China (98°25′–100°02′ E, 24°08′–25°51′ N). The entire study area has a subtropical plateau climate, with the local annual temperature difference being slight but the daily temperature difference being significant. The average annual temperature is 15.5 °C, the average temperature of the coldest month is 8.2 °C, and the average temperature of the hottest month is 21 °C. The highest precipitation occurs over the western and southern areas. The terrain is high in the northwest and low in the southeast. The city is bordered by Dali, Lincang, the Salween River, and the Dehong Dai and Jingpo Autonomous Prefecture. The city spans three major river systems, namely, the Lancang, Salween, and Longchuan. The region is rich in terms of land, forest, and mineral resources and natural gas reserves; moreover, it is a hub for hydroelectric power generation and has several tourist attraction spots. The types of cultivated land are mainly dryland and a small number of paddy fields. The cultivated land in the study area is shown in Figure 1. 

**Figure 2:**
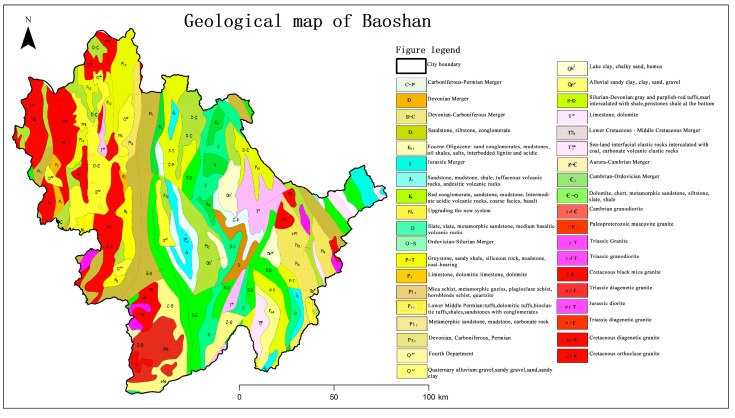
Geological maps of the study area.

Many experts and scholars investigated the origin of the parent rock mass in Baoshan City and found severe PTE enrichment in soils around the Baoshan mining area. [17,32] The geology of Baoshan City mainly includes carbonate rocks and basalt parent materials; the geological composition of the full study area is shown in Figure 2. In addition, the mining of the rich mineral resources in Baoshan has caused contamination and parent material enrichment of the soil nearby. Moreover, the recent economic development of Baoshan City has driven the expansion of non-ferrous metal smelting, real estate development, animal husbandry, old and outdated production facility construction, and desulfurization, resulting in contaminated farmland.

### 2.2. Sample Collection and Preparation and Quality Control

We randomly collected 152 topsoil samples (at 0–20 cm depth) from agricultural land in Baoshan in August 2022, using a five-point sampling method with plum blossom. Moreover, 1 kg of soil samples was collected by mixing and dividing the samples into four parts. The sampling points were located by GPS. All the soil samples were stored in plastic bags and transported to the laboratory for air drying and removing plant roots, residues, and visible invaders. Samples were passed through a nylon sieve with a 2 mm pore size to remove soil samples greater than 2 mm. Then, the sieved samples were ground and passed through a nylon sieve with a 0.149 mm pore size (100 mesh), mixed, and prepared for analyses of the soil PTEs, Cd, Pb, Cu, Zn, and As. Table 1 shows the specific methods used for the determination of potentially toxic elements. A flame atomic absorption spectrometer and graphite furnace atomic absorption spectrometer (AA6880, Shimadzu, Kyoto, Japan) and atomic fluorescence spectrophotometer (AFS-230E, Haikou Instruments, Beijing, China) were used for element detection. National standard soil samples (GSS-25) were used for quality control. The test samples were analyzed three times each, and their relative standard deviation was ≤5%. Blank samples were tested for the determination of each heavy metal. Heavy metal standard solutions were used for each batch of sample reagents, and the element recovery rate was 90–110%.

### 2.3. Pollution Assessment

#### 2.3.1. Pollution Factors

The pollution level of the study area was evaluated by pollution factor evaluation [33,34,35].

(1) $$\mathrm{PF}=\frac{C_{i}}{B_{i}}$$

where *C_i_* is the soil PTE concentration (mg/kg), and *B_i_* indicates the corresponding background values of soil PTEs in Yunnan Province (mg/kg) (Cd 0.22, Pb 40.6, Cu 38.38, Zn 89.7, As 18.4). The PF values’ environmental indices could be divided into four classes to interpret the pollution levels of soil PTEs (Table 2) [36].

#### 2.3.2. Geoaccumulation Index

The geoaccumulation index (I_geo_) was developed by Muller (1969) to assess the level of heavy metal and metalloid elements in the sediment by comparing the status of the current concentration with the pre-industrial level. This method assesses heavy metal pollution related to anthropogenic activity, as well as the different rock geology regions, and the natural formation of heavy metal pollution [37].

(2) $$I_{\mathrm{geo}}=\log_{2}\frac{C_{n}}{1.5\times B_{n}}$$

where *C_n_* is the soil PTE concentration (mg/kg), and *B_n_* is the corresponding background value of soil PTEs in Yunnan Province (mg/kg). The obtained I_geo_ values were classified into seven groups based on Categories (Table 3).

### 2.4. Receptor Models

Based on the receptor model, the contributions of various soil pollution sources were analyzed quantitatively by mathematical method according to the source and concentration of the receptor. Employing PMF, UNMIX, and APCS-MLR, several factors were extracted and identified according to source types, and their contributions were estimated.

#### 2.4.1. APCS-MLR

Principal component analysis is a multivariate statistical analysis method that selects a small number of important variables by linear transformation. Using the linear combination of the original variables after standardization to form the principal component, PCA model can be transformed into several complementary and related comprehensive indexes. The method is used widely in, e.g., demographics, quantitative geography, molecular dynamics simulation, mathematical modeling, and mathematical analysis. The APCS-MLR model was proposed by Thurston [38]. After the data were standardized, the principal component factor was transformed into an absolute principal component score (APCS) by factor analysis. Subsequently, multiple linear regression analyses were performed to determine the content of each selected heavy metal, and the contribution rate of each factor to the pollution source was further calculated.

(1) Standardizing raw data
  (3) $$Z_{ij}=\frac{C_{ij}-\bar{C_{i}}}{\sigma_{i}}$$
  where *Z_ij_* is the standardized factor score, and *C_ij_* is the soil PTE*_i_* concentration (mg/kg). $\bar{C_{i}}$ is the average soil PTE*_i_* concentration (mg/kg); $\sigma_{i}$ is the standard deviation of soil PTE*_i_* (mg/kg).
(2) Introducing a factor with a concentration of 0
  (4) $$Z_{i0}=\frac{0-\bar{C_{i}}}{\sigma_{i}}$$
  where APCS for each heavy metal element is obtained using *Z_ij_* − *Z_i_*_0_. Using the obtained APCS, multiple linear regression analysis is conducted to obtain the regression coefficient:
  (5) $$C_{i}=b_{i0}+\sum_{p=1}^{p}\left(b_{pi}\times APCS_{p}\right)$$
  where *B_i_*_0_ is the constant obtained from multivariate linear regression; *b_pi_* is the regression coefficient of source *p* to the soil PTE; and *b_pi_* × *APCS_p_* is the source contribution to *C_i_*.

#### 2.4.2. PMF

Positive matrix factorization (PMF) analysis, proposed by Paatero et al. [39], is a source analysis method used widely by the United States Environmental Protection Agency (USEPA). The method is often used in sediment, atmospheric, and soil contamination source analyses. The PMF model is least squares through multiple iterations to minimize the objective function Q for obtaining the optimal factor matrix and source profile.

(6) $$Q=\overset{n}{\underset{i=1}{\sum }}\overset{m}{\underset{j=1}{\sum }}{\left(\frac{x_{ij}-\sum_{k=1}^{p}G_{ik}F_{kj}}{u_{ij}}\right)}^{2}\to Q=\overset{n}{\underset{i=1}{\sum }}\sum_{j=1}^{m}{\left(\frac{e_{ij}}{u_{ij}}\right)}^{2}$$

where *x_ij_* is the content of the *j* heavy metal element in the *i* sample; *G_ik_* is the contribution of source *k* to the *i* sample; *F_kj_* is the content of the *j* heavy metal element in the *k* source; *u_ij_* is the measured uncertainty (mg/kg); and *e_ij_* is the model uncertainty. The sample chemical type uncertainty file is calculated as follows:

(7) $$u_{ij}=\left\{\begin{array}{c} \;\frac{5}{6}\times MLD,x_{ij}<MLD\\ \sqrt{\left(x_{ij}\times {\left.Error\;Fraction\right)}^{2}+{\left(MLD\times 0.5\right)}^{2}\right.}\;,\;x_{ij}>MLD \end{array}\right.$$

where *C_ij_* is the concentration of the *j* sample chemical type of the *i* sample, and MDL is the species-specific method detection limit. The error fraction is a percentage of measurement uncertainty.

#### 2.4.3. UNMIX Model

The UNMIX is a receptor model that is based on the pollutant concentration. The major sources and contributions of hand-held pollutants can be obtained directly through extremes. The UNMIX6.0 software be identified to Klstrong in data quality that signal-to-noise (S/n) was more than 2, and the fitting concentration was more than 0.8. Simple software operation, the results do not need their own analyses but cannot evaluate the source component, contribution rate, or contribution value. We used Equation (8), recommended by the USEPA, for performing source apportionment for potentially toxic elements.

(8) $$C_{ij}=\overset{p}{\underset{l=1}{\sum }}\left(\overset{p}{\underset{k=1}{\sum }}U_{ik}D_{kl}\right)V_{lj}+\varepsilon_{ij}$$

where *U*, *D*, and *V* are the *np* diagonal matrix, *p × p* diagonal matrix, and *p × m* matrix, respectively. *ε_ij_* is the error term that contains the variability of *C_ij_* and excludes the first major component *p*.

### 2.5. Data Treatment with Computer Software

The data was pre-processed using Excel 2010 software. The SPSS^®^ Statistics 23.0 (Armonk, NY, USA) software has performed normal distribution test and statistical analysis of data of potentially toxic elements in soil. The figures were drawn by Origin 2021. The geostatistical analyst tools data exploration tool set analysis was performed by ArcGIS 10.6. The sampling point distribution map and heavy metal pollution spatial distribution feature map were drawn by ArcGIS 10.6. The analysis of PMF was conducted with PMF 5.0.

## 3. Results and Discussion

### 3.1. Pollution Characteristics of Soil PTEs 

#### 3.1.1. Soil PTE Concentration

**Figure 3:**
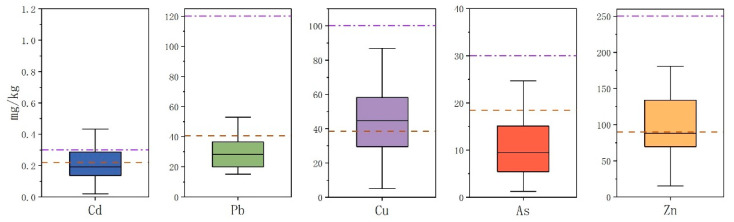
Box plot of soil PTEs in the study area. The red line of the box plot of soil PTE content represents the background value, and the purple line represents the risk screening value.

Descriptive statistics for Cd, As, Pb, Cu, and Zn in soil PTEs are shown in Table 4. Compared with the background values for Yunnan Province [40], the average concentrations of PTEs Cd (0.28 mg/kg), Cu (47.59 mg/kg), and Zn (100.46 mg/kg) in soils were all higher, and the median values were 1.25, 1.24, and 1.12 times higher, respectively, than the corresponding regional background values. The average concentrations of soil PTEs did not exceed the national soil pollution risk screening values (GB15618-2018), indicating an overall low degree of pollution in the cultivated land of the study area. The box plot in Figure 3 shows the relationship between single soil sample and background values, as well as the risk screening value in Yunnan Province. The Cd content in 25–75% of samples was higher than the background and risk screening values. The contents of the potentially toxic elements As, Pb, Cu, and Zn in 25–75% of the samples were higher than the background values, and the contents of Cu and Zn in 54.6–48.6% of samples were higher than the background values.

In 35.53, 21.05, 54.61, 48.68, and 20.39% of the soil samples, the Cd, Pb, Cu, Zn, and As contents, respectively, exceeded the background values. The Cd, Pb, Cu, and As contents exceeded the risk screening values in 21.34, 2.63, 5.26, and 5.26% of the samples, respectively. These findings indicate the contamination of arable land in the study area and thus emphasize the need for further analysis of the contamination status.

The coefficient of variation (CV) reflects the degree of soil disturbance caused by anthropogenic activities. Table 4 shows the high variability of species elements, with variabilities of 0% < CV ≤ 12%, 12% < CV ≤ 31%, and >31% indicating low, moderate, and high variabilities, respectively [41]. The variations of Cd and As were 91.3 and 81.5%, respectively, indicating high degrees of variation and disturbance by anthropogenic activities. These activities include smelting and sugar production and plant fuel burning and sewage discharge causing Cd and As soil pollution. As Baoshan is located in the Cu–Pb–Zn belt, mining of these metals causes severe Cd, As, Cu, Zn, and Pb pollution of the surrounding farmland [42]. However, agricultural activities also lead to increasing Cd and As contents in farmland [43]. 

Relatively low variations were observed for Zn, Pb, and Cu. Both carbonate and basalt parent materials are developed in the Baoshan area, and soils with basalt parent materials such as granite are rich in Cd, Cu, and Zn. Soils derived from carbonate rocks are rich in Cd, Pb, Cu, and Zn, and their natural genesis results in high Zn and Cu contents and low coefficients of variation [44,45]. However, the Zn and Cu contents of the samples (48.6 and 54.6%, respectively) were higher than the background values. Duan et al. [46] found that improper agricultural practices could lead to an excess of Cu and Zn in soils, and mining could cause Zn, Cu, and Pb pollution. The pollution levels in the overall study area were low, but the soil PTE coefficient of variation was high, which could be owing to the fact that soil pollution is complex and can be caused by a combination of natural and anthropogenic factors. Accordingly, a policy for preventing further risk and a control strategy should be adopted, implying that the source of regional pollution must be identified to facilitate timely prevention and control.

#### 3.1.2. Assessment of Soil PTE Pollution

**Figure 4:**
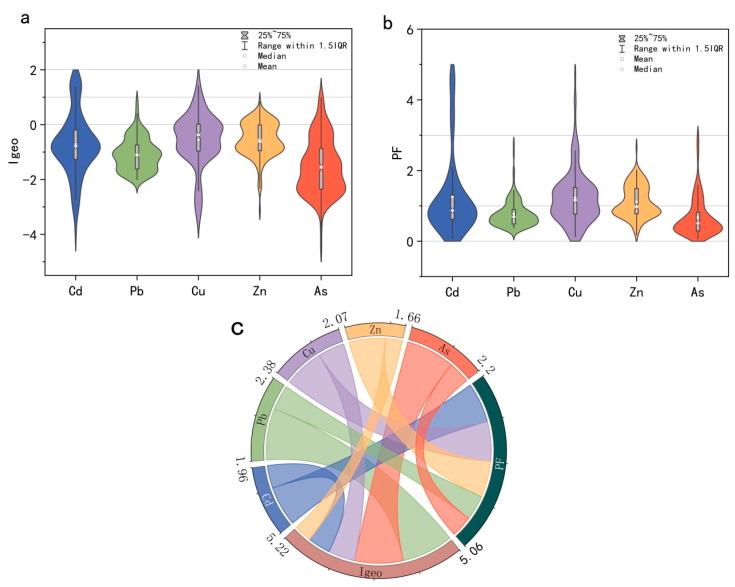
Violin with box plot of soil PTEs in the study area (**a**) I_geo_, (**b**) PF, and (**c**) the relationship between soil PTEs and environmental indices.

To further advance our understanding of the extent of soil contamination, the PTEs in the study area were assessed using I_geo_ and PF, as shown in Figure 4. The results showed that the mean I_geo_ median and mean values of PTEs in five soils in the study area were all < 0, indicating non-pollution (Figure 4a); however, the soil PTE, Cd (10.53%), Pb (3.29%), Cu (23.68%), Zn (23.68%), and As (7.24%) samples showed weak to moderate pollution. A small proportion of soil was moderately polluted by PTEs (Cd, 11.8%; Cu, 2.6%). Similarly, as shown in Figure 4b, the average PF values of five PTEs in soils in the study area were all less than 3, indicating that the overall pollution in the study area was moderate. The average PF value of Cd (1.25) was the highest, followed by those of Cu (1.24), Zn (1.12), Pb (0.77), and As (0.67). The PF median values of <1 for Cd and Zn indicate moderate pollution, but the mean values of >1 for Cd and Zn indicate low pollution implying that most cultivated areas were slightly polluted, whereas other areas were weakly to moderately contaminated, with PTE contributions of 23.68, 21.05, 51.97, 48.68, and 20.39% for Cd, Pb, Cu, Zn, and As, respectively. A small part of the soil showed moderately contaminated, and the values for Cd (11.84%) and Cu (2.63%) indicated severe pollution from these elements in the farmland soil.

The relationship between the two pollution indices and soil PTEs is shown in Figure 4c. As the values of the five types of soil PTEs were less than 0, the smaller the PTE, the greater would be its proportion. Therefore, the largest contribution of soil PTEs to I_geo_ was of Zn and Cd, followed by that of Cu, As, and Pb. The greatest contribution of PTEs to PF was of Cd and Cu, followed by that of Zn, Pb, and As. As the results of the various pollution assessment methods differed, neither the degree of soil PTE pollution nor the pollution sources could not be judged directly. Therefore, assessing soil PTE pollution and pollution concentrations must be combined with spatial analyses to gradually explore the source of PTEs for related risk assessment.

### 3.2. Spatial Distribution of Soil PTEs

**Figure 5:**
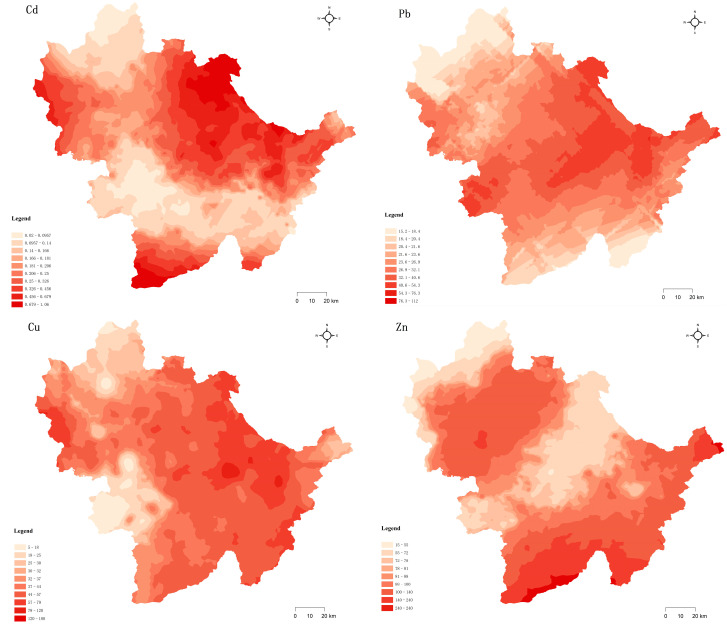

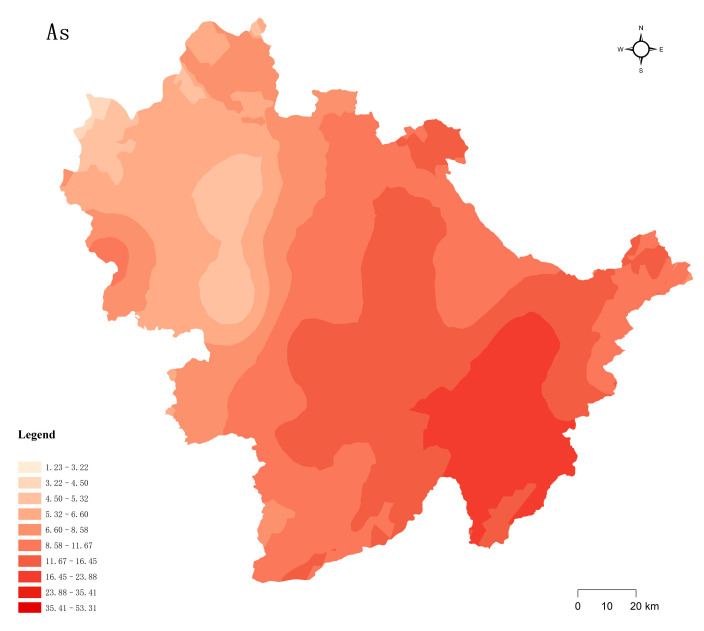
Spatial distribution of soil PTEs in the study area.

Analyses of the distribution of soil PTEs could provide information on the extent of soil pollution in the study area and improve our understanding of the sources of soil PTEs [47]. The data were interpolated using ArcGIS with the Kriging method, as shown in Figure 5. The distribution of Zn and Cu in soil was partly similar, with the high concentration area located near the northwest of China, where numerous coal mines are situated. Tu et al. [48] found that the development of the coal industry led to Zn and Cu pollution in the surrounding farmland. High concentrations of Cd, Pb, and Cu were found in the northeast of China, with the high Pb values being closer to the South, which are the main concentration areas of cities and towns. Lai et al. [49] indicated that automobile exhaust gas was the main source of Pb pollution. The high Cd and Cu value area is near the North. The investigation showed tungsten and copper deposits in the area, which are mostly located in the high mountain area. As and Zn were mainly concentrated in the southeast, with the spatial distribution characteristics of potentially toxic elements in soils being similar. Hu et al. [50] showed that the use of herbicides, chemical fertilizers, and animal manure could cause As and Zn pollution in soils. Pollution from Cu is distributed widely, and the other four soils (Cd, Zn, Pb, and As) exhibit concentrated distributions of PTEs. Therefore, step-by-step exploration of the sources of PTE contamination was necessary to analyze the source of soil PTE pollution based on a combination of receptor models and geostatistics.

### 3.3. Source Apportionment of Soil PTEs

#### 3.3.1. Multivariate Statistical Analysis

**Figure 6:**
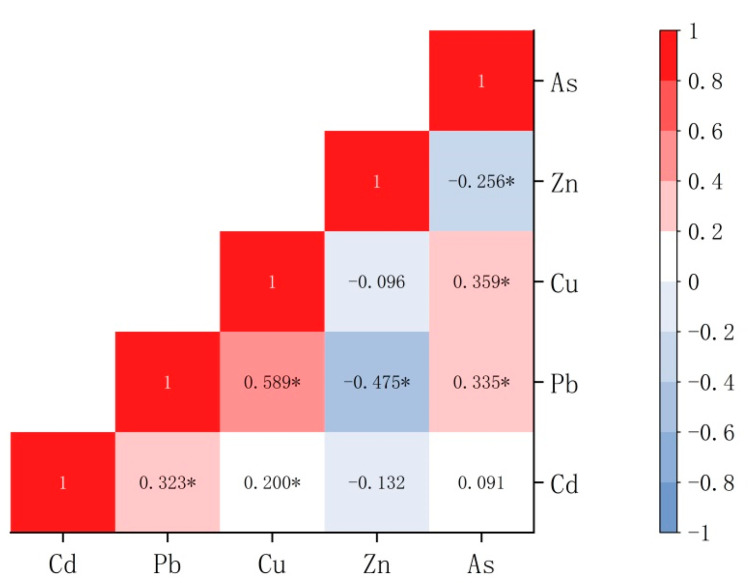
Correlation analysis for soil PTEs. (* indicates significant correlation.)

Based on the correlation between PTEs, we used Pearson correlation analysis to determine whether the sources of soil PTEs were consistent [51]. A significant positive correlation between the elements indicates a similar source between the elements; a significant negative correlation between the elements indicates a difference in source between the elements [52]. The results of the correlation coefficient analysis are shown in Figure 6. Positive correlations were found between Pb–Cd, Cd–Cu, Cu–Pb, As–Pb, As–Cu, and As–Pb, and negative correlations were found between Pb–Zn and As–Zn. The Cu–Pb correlation coefficient was 0.589 (*p* < 0.01), indicating that Cu–Pb comes from the same pollution source, possibly industrial or traffic pollution. The following observations were made: (1) Pb is correlated with As, Cu, and Cd, which may be owing to the high proportion of Pb in different pollution sources; (2) the sources of Cd, Pb, Cu, and Zn could be similar; (3) Zn–Pb and As–Zn were correlated negatively with each other, indicating that they could have the same source but in opposite proportions. The source of each element and the correlation between them could be verified further with PCA.

#### 3.3.2. APCS-MLR Model

**Figure 7:**
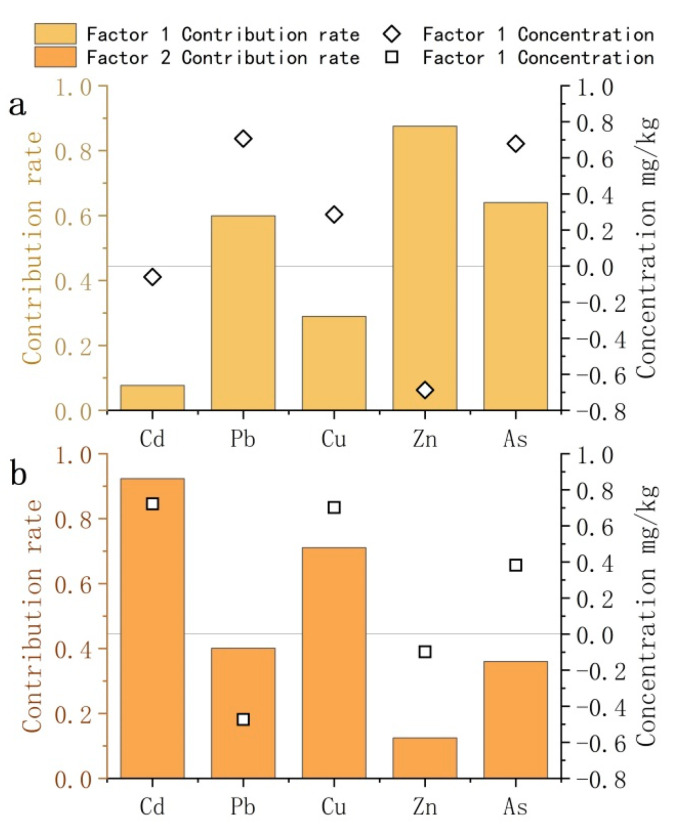
Source profiles and source concentrations of soil potentially toxic elements from PCA. ((**a**) is the source profiles and source concentrations of soil potentially toxic elements for factor 1; (**b**) is the source profiles and source concentrations of soil potentially toxic elements for factor 2).

We used SPSS Statistics 23.0 to analyze the soil heavy metal concentration. The extraction characteristics of the first two principal components were more than 1; the Kaiser–Meyer–Olkin measure of sampling adequacy and Bartlett values were 0.53, and the Bartlett spherical test *p* value was 0.00 (*p* < 0.05). These results showed a strong correlation among the potentially toxic elements, which could be analyzed using PCA. The cumulative contribution of variance was 58.25%, which could explain most of the information on the soil PTEs. Potentially toxic elements with a higher factor load under the same principal component have the same origin [53,54]. The first principal component (PC1) was Pb, Zn, and As, the variance contribution was 30.35%, and Zn was the main factor. Although the average values of Pb and As were lower than the background values in Yunnan Province, the samples of Pb (21%) and As (20%) sites exceeded the background values. As shown in Figure 7, the areas with high Pb concentrations were mainly located close to the Longyang District towns and cities, with the As concentrations deriving mainly from fertilizers, fossil fuels, and anthropogenic activities [55]. Investigating the mineral distribution in Baoshan showed the presence of numerous iron and coal mines in the region, the mining of which results in Pb, Zn, As, and Cu pollution [56]. Agricultural activities cause Zn and Cu pollution because of unreasonable fertilizer use, excessive application of conditioning agents, and agricultural machinery activities [57,58]. Therefore, Factor 1 could be considered anthropogenic pollution.

The second component (PC2), with a higher driving, included Cd and Cu, of which the variance contribution was 27.91%, with Cd being the main factor. Significant correlation was found between Cd and Cu, indicating that both pollutants were likely derived from the same source. Moreover, Baoshan is located on carbonate and basalt parent material, and carbonate parent material can weather Cd, Cu, Pb, Zn, and As. The basalt matrix could be divided into Cd, Cu, and Zn [59], and Cu and Cd could be differentiated from the mixed carbonate matrix [60]. Yunnan Province is characterized by a complex geological structure and rich metal deposits. The main distribution area of Cd and Cu is alpine cultivated land. Therefore, Factor 2 could be artificially induced as a natural cause.

The contribution rates of soil PTEs used to calculate the APCS score and the APCS-MLR receptor model of each soil PTE were obtained by using regression analysis (Figure 7). The main contributing factors were As, Cu, and Pb [61], which could be considered as agricultural pollution, with the main contributing factors being Cd, Zn, and Cu [62]. These results suggested that Factor 2 was a natural cause, and Factor 3 was the main contributing factor of Pb, Cd, and Zn, i.e., industrial and traffic pollution. Our results were consistent with those of [63].

#### 3.3.3. PMF Model

**Figure 8:**
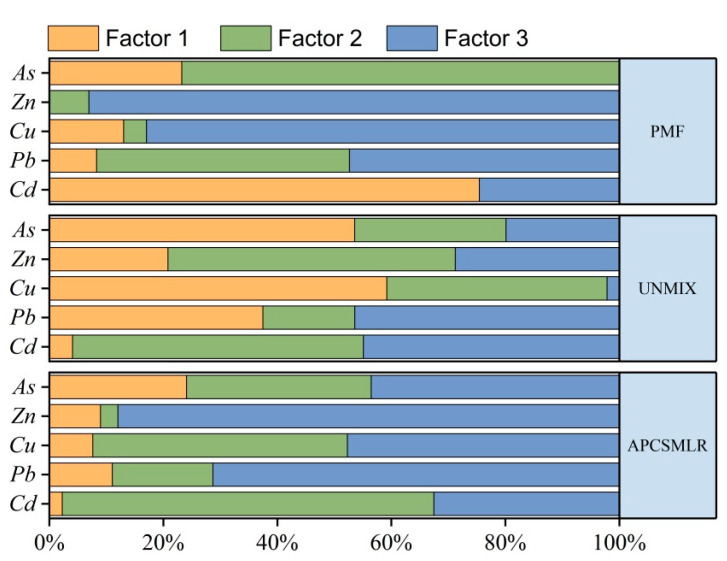
Source contribution of each factor derived from PMF, UNMIX, and APCS/MLR.

Employing the PMF model, we determined that the signal-to-noise ratio (S/N) of all chemicals was greater than the combined Q value, the resulting source profile, and the scale residuals. The model was run 20 times, resulting in the identification of three sources, as shown in Figure 8. The main contributing factors to Factor 1 were Cd, As, and Cu [64], whereas those to Factor 2 were As and Pb [65]. The results showed that As and Pb pollution could be caused by the application of chemical fertilizers and pesticides in agriculture, i.e., As agricultural pollution. These results were consistent with the analysis results of Han et al. using the PMF model [66]. The main contributing factors of Factor 3 were Zn, Cu, and Pb, which could be considered industrial and traffic pollution. The results were consistent with those of Wang et al. [65].

#### 3.3.4. UNMIX Model

We used UNMIX 6.0 software to analyze the sample concentration data. The results showed that Min-Rsq was 0.82, i.e., higher than the threshold of 0.8, and S/N was 2.21, greater than the system requirement maximum threshold of 2. Therefore, the approach was considered successful, and three sources could be detected, as shown in Figure 8. Factor 1 was mainly contributed to by Cu, As, and Pb [67], with the assumption being that Factor 1 is agricultural pollution. The main contributing factors to Factor 2 were Cd, Zn, and Cu [68], with Factor 2 considered natural causes. The main contributing factors to Factor 3 were Pb, Cd, and Zn, which could be considered industrial and traffic pollution. These results were consistent with those of Luo et al. [69].

### 3.4. Model Evaluation

**Figure 9:**
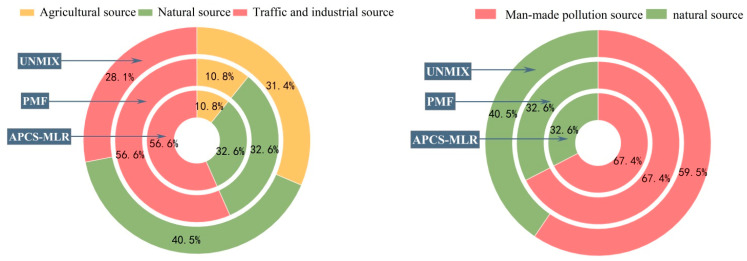
Estimated average source contribution (%).

The results of the three models were consistent. As shown in Figure 9a, the main sources of pollution derived from industrial activities and traffic, followed by natural causes and agricultural activity. Clearly, the pollution originated from human activity. Combining the results from the three receptor models enabled us to compile the information more comprehensively. As shown in Figure 9b, the main source of pollution in the cultivated land was disturbance by humans, which severely affected the quality of the land. Combined with Figure 7, Figure 8 and Figure 9, the four models identified three common source categories other than PCA, namely, natural sources (35.23%), agricultural sources (17.67%), and industrial and transportation sources (47.12%). The difference between the model results was that UNMIX yielded a low resolution of industrial and transportation sources and a high resolution of natural and agricultural sources, whereas APCS-MLR and PMF and resolution of the same contribution rate. In addition to UNMIX, the APCS-MLR and PMF models revealed that the agricultural source was dominated mainly by As, whereas the natural source was dominated primarily by Cd. We employed the UNMIX model to analyze the Cu with the highest contribution rate to As, with As being considered the main factor in agricultural pollution. The results of the other three models accounted for a substantial proportion of Zn and Pb in the analysis of industrial and traffic pollution. The potential variables could not be estimated by PCA, and the negative values limited the results of the analysis. Although APCS-MLR evolved from PCA, negative numbers remained in the calculation process. In contrast, UNMIX is a simple model that does not require setting the number of pollution sources and well explains the species concentrations [70]. Employing PMF, we could estimate the error at each site. The reasonable treatment of missing and imprecise data is used widely in soil and air pollution analyses; however, the results are affected by uncertainties of the data and model structure [29]. Three source apportionment models could be used to quantitatively identify pollution, with the contribution rate being the only difference [71,72]. Accordingly, an error that could be caused by employing a single model could be counteracted by the comparative analysis of several receptor models [73,74].

## 4. Conclusions

The pollution characteristics and source contributions of PTEs in cultivated soils in Baoshan were investigated, and three receptor models were used to analyze and compare the pollution sources in the study area. The concentrations of Cd, Cu, and Zn in soil were higher than the corresponding background values in Yunnan province. According to the I_geo_ and PF values, the cultivated soil was polluted to different degrees. In particular, pollution from Cd and Cu was severe. The analytical results of the APCS-MLR, PMF, and UNMIX models indicated that As and Pb pollution was caused by agricultural activities, with As pollution deriving mostly from the improper use of agricultural chemicals, together with Cu and Zn pollution. Moreover, the use of agricultural machinery in the region led to Pb pollution. The natural source is mainly caused by the elements Cd and Cu. As the study site is a carbonate-weathering area, Cd is the most significant controlling factor. Automobile exhaust emissions were the main source of Pb and Zn pollution. The emissions of Cu and Cd were the main source of Zn pollution. Overall, 64.76% of the pollution in Baoshan derived from agriculture, of which 47.12% of the pollution derived from industrial transport. Accordingly, this local study should pay more attention to the pollution by industrial production, and more attention is required to mitigate pollution caused by industrial production and traffic emissions.

## Figures and Tables

**Table 1:** Detection methods.

Test Items	Detection Method	GB Number	Detection Limits
Copper (Cu) Zinc (Zn)	Furnace atomic absorption spectrophotometry	GB/T17138-1997	0.01 0.5
Cadmium (Cd) lead (Pb)	Graphite furnace atomic absorption spectrophotometry	GB/T17141-1997	0.01 0.2
Arsenic (As)	Atomic fluorescence Spectrophotometer	GB/T22105	0.02

**Table 2:** PF with different classifications.

PF	Level of Pollution
PF ≤ 1	Low pollution
1 < PF ≤ 3	Moderate pollution
3 < PF ≤ 6	Considerable pollution
PF > 6	Very high pollution

**Table 3:** Geoaccumulation index with different classifications.

Level	I_geo_	Level of Pollution
Ⅰ	I_geo_ ≤ 0	Not to weakly contaminated
Ⅱ	0 < I_geo_ ≤ 1	Weakly to moderately contaminated
Ⅲ	1 < I_geo_ ≤ 2	Moderately contaminated
Ⅳ	2 < I_geo_ ≤ 3	Moderately to strongly contaminated
Ⅴ	3 < I_geo_ ≤ 4	Strongly contaminated
Ⅵ	4 < I_geo_ ≤ 5	Strongly to extremely contaminated
Ⅶ	I_geo_ ≥ 5	Extremely contaminated

**Table 4:** Descriptive statistics of soil PTEs in the study area (mg/kg). Min = minimum value; Max = maximum value; Med = median value; AM = arithmetic mean; SD = standard deviation; CV = coefficient of variation; Sk = skewness; Ku = kurtosis; k-s test = Kolmogorov–Smirnov test; BG = background values of Yunnan Province (CNEMC, 1990); RSV = risk screening values of the soil PTEs (GB15618-2018).

PTEs	Min	Max	Med	AM	SD	CV (%)	Sk	Ku	k-s Test	BG	RSV
Cd	0.02	1.06	0.19	0.28	0.251	91.3	1.921	2.806	0	0.22	0.3
Pb	15.15	112	28.14	31.42	15.28	49.0	2.168	7.119	0	40.6	120
Cu	5	181.3	44.6	47.59	29.129	61.2	1.575	4.018	0	38.38	100
Zn	15.44	239	87.74	100.46	39.664	39.5	0.493	−0.177	0	89.7	250
As	1.23	54.46	9.39	12.36	10.076	81.5	2.016	4.63	0	18.4	30

## Data Availability

Not applicable.
